# A Descriptive Qualitative Study of Patient and Carer Perspectives on the Acceptability of Transcatheter Aortic Valve Implantation

**DOI:** 10.1111/jan.70180

**Published:** 2025-09-03

**Authors:** Nicola Straiton, Robyn Gallagher, Janice Gullick

**Affiliations:** ^1^ Nursing Research Institute, St Vincent's Health Network Sydney, St Vincent's Hospital Melbourne Australian Catholic University Sydney New South Wales Australia; ^2^ School of Nursing, Midwifery and Paramedicine Australian Catholic University Sydney New South Wales Australia; ^3^ Faculty of Medicine and Health, School of Nursing University of Sydney Sydney New South Wales Australia

**Keywords:** acceptability, aortic stenosis, carer, implementation science, patient, transcatheter aortic valve implantation

## Abstract

**Aim:**

To provide a structured analysis of the acceptability of transcatheter aortic valve implantation to support clinical conversations, decision making and recovery for older adults with aortic stenosis and their carers.

**Background:**

While transcatheter aortic valve implantation is an effective treatment for heart valve disease, its acceptability to patients and caregivers remains unclear. Understanding the acceptability of clinical procedures is key for influencing patient engagement in self‐care and guiding the information and support patients and carers need.

**Design:**

A descriptive, qualitative study used deductive content analysis, guided by Sekhon's Theoretical Framework of Acceptability.

**Methods:**

Participants included 18 aortic stenosis patients (mean age 84.2 ± 4.1 years) and 8 carers from three Australian metropolitan hospitals (2018–2020). Semi‐structured interviews were conducted 4–6 months post–TAVI and transcribed verbatim. Analysis used Sekhon's Theoretical Framework of Acceptability across three temporal zones, with deductive coding examining affective attitude, burden, ethicality, intervention coherence, opportunity costs, perceived effectiveness and self‐efficacy.

**Results:**

Participants described high prospective, concurrent and retrospective acceptability of transcatheter aortic valve implantation. Perceived prospective acceptability framed the procedure as lifesaving. Peri‐operatively, participants found the procedure simple, low‐risk and minimally disruptive, ensuring high concurrent acceptability. Post‐procedure, patient participants described a slow but gradual return to normal, growing confidence and a reengagement with their valued pastimes. The absence of structured rehabilitation advice led to self‐designed recoveries and uncertainty about safe limits.

**Conclusion:**

Transcatheter aortic valve implantation was perceived as a highly acceptable intervention that helped this group of mostly older adults achieve their personal goals.

**Relevance to Clinical Practice:**

Despite the minimally invasive nature of transcatheter aortic valve replacement, optimising recovery and rehabilitation requires a holistic approach that addresses both clinical needs and patient goals.

**Patient and Public Contribution:**

None in the conceptualisation or design.

## 1 Introduction

Aortic stenosis (AS) is the most common heart valve disease in the developed world, with the prevalence increasing alongside the ageing population (Chatterjee et al. 2023; Joseph et al. 2017). In higher‐income countries, AS is the third leading cause of cardiovascular death and in 2019 there were an estimated 9.4 million cases of calcific aortic valve disease globally, and 102,700 associated deaths (Roth et al. 2020; Yadgir et al. 2020).

AS is often characterised by thickening and reduced mobility of the aortic valve leaflets, which impedes blood flow from the left ventricle to the aorta and to the rest of the body, often impairing cardiac output. Over time calcification worsens and the valve outlet begins to narrow, resulting in severe AS with progressive symptoms of heart failure, primarily dyspnoea, but also angina and syncope. Once AS becomes severe and the symptom burden (particularly breathlessness) increases, there is a correspondingly poor prognosis with a mortality rate of around 50% at 2 years and 80% at 5 years (Czarny and Resar 2014; Sitges et al. 2021). Consequently, the natural course of AS without treatment is eventual death. AS symptoms can also seriously impact a person's ability to function and manage their lives, which can impact their mental health, leading to increased stress, anxiety and depression (Picou et al. 2022). Family and friends are frequently relied upon to provide tangible and emotional support to manage basic self‐care tasks such as cooking, mobility or helping to make sense of the disease, treatment options and prognosis (Ingle et al. 2022). Lack of this support is strongly correlated with depression, and poorer morbidity and mortality outcomes in cardiovascular disease (Su et al. 2018; Tan and Wang 2019).

Understanding these personal and contextual factors is essential, as they influence how individuals interpret their diagnosis, assess treatment options, engage with healthcare, and ultimately shape their recovery trajectories and goals of care following interventions (Schuler et al. 2025).

## 2 Background

Aortic valve replacement (AVR) stands as the recommended intervention for severe AS, either by surgical aortic valve replacement (SAVR) with open‐heart techniques or the less invasive transcatheter aortic valve implantation (TAVI) (Vahanian et al. 2021). The first TAVI procedure was performed globally in 2002 as part of a clinical trial, with Australia's first procedure taking place in 2008. Initially, TAVI was accessible in Australia only through compassionate use programmes or clinical trials. Broader Australian adoption began following the introduction of Medicare Benefits in November 2017 (Gray and Sarathy 2022).

TAVI is particularly favoured for individuals aged 80 and above, those with complex co‐morbidities or experiencing severe frailty, or those at a prohibitive risk of mortality from SAVR, demonstrating clear improvements in mortality and morbidity. Several studies and systematic reviews demonstrate enhanced survival rates post‐TAVI and improvements in functional capacity and health‐related quality of life for AS patients.

However, these are not universally experienced and may be less pronounced among those who are frail or burdened by multiple chronic conditions (Lunardi et al. 2022). Despite over 1.5 million TAVI procedures performed globally, limited research exists on how patients with AS and their families evaluate the acceptability of TAVI—how they weigh the potential benefits and risks in light of their health status, personal values and life circumstances. This process includes assessing the procedure's impact on quality of life, balancing its effectiveness against frailty and comorbidities, and determining whether it aligns with individual treatment goals.

Notably, a study on patient‐centred benefit–risk analysis found that patients often prioritised attributes such as reduced invasiveness and faster recovery—factors linked to quality of life—over the more established durability and lower pacemaker risk associated with SAVR (Ting et al. 2014). Shared decision‐making can be further complicated by a disconnect between clinicians' perceptions—that patients prefer to defer to medical recommendations—and growing evidence that many patients wish to participate meaningfully in decisions about their care (Lauck et al. 2021). Given the progressive nature of AS, people's openness to particular interventions may evolve over time, requiring patients and carers to continuously reassess their options and decisions in the context of changing health state and personal priorities (Kalogeropoulos et al. 2022).

Healthcare intervention acceptability has been defined as: ‘a multi‐faceted construct that reflects the extent to which people delivering or receiving a healthcare intervention consider it to be appropriate, based on anticipated or experienced cognitive and emotional responses to the intervention’ (Sekhon et al. 2017). In Sekhon's Theoretical Framework of Acceptability, factors that influence perceived acceptability of healthcare interventions include the intervention's appropriateness in addressing the clinical problem, alignment with individual values and beliefs, suitability to lifestyle and convenience and overall effectiveness in managing the clinical problem. Lack of intervention acceptability can negatively impact patient engagement with healthcare services or treatments, leading to poorer health outcomes, and may subsequently influence the scalability and sustainability of a healthcare intervention (Chegini et al. 2020; Klaic et al. 2022).

Understanding how individuals with aortic stenosis and their carers perceive the acceptability of TAVI, alongside their lived experiences, provides vital insights into the real‐world challenges and expectations surrounding the procedure. This is an often‐overlooked aspect as procedural volumes rise to meet the growing disease burden. While TAVI demonstrates benefits in survival, function and quality of life, these outcomes vary, especially among frail patients and those with multiple co‐morbidities. Exploring acceptability helps identify factors that shape patients' and carers' willingness to undergo, adhere to, and engage with the procedure and follow‐up care. This understanding is essential for informing the scalability and sustainability of TAVI programmes, as it directly impacts their growth, continuity and integration into existing and new healthcare settings (Milat et al. 2020).

The aim of this study was to explore the lived experiences of AS and understand how personal perspectives, health and life contexts may influence the acceptability of TAVI for patients and carers. These perspectives will encompass acceptability of pre‐operative information, the operative procedure, the recovery process and engagement with services that promote rehabilitation.

## 3 Methods

### 3.1 Design

This is a descriptive qualitative study. Descriptive qualitative research frequently draws on theories, models and concepts relevant to a discipline, or drawn from sociology, which then provide the research framework. In such cases, data analysis is guided by concepts from the theoretical framework and results in recognition of recurring patterns or categories that cut through the data and help to further refine the theoretical framework as an explanation for findings within the chosen context (Caelli et al. 2003).

### 3.2 The Theoretical Model

Sekhon's Theoretical Framework of Acceptability (Sekhon et al. 2017) draws on foundational concepts of acceptability which incorporate individual and societal perspectives, values, attitudes and judgements perceived or expressed by patients, carers and clinicians (Sidani et al. 2009; Staniszewska et al. 2010). This framework has been widely applied to evaluate the acceptability of healthcare interventions, including in areas such as joint replacement surgery, medication error reduction and venous access management in patients with complex liver disease (Paynter et al. 2023; Laing et al. 2022; Sheils et al. 2020).

**FIGURE 1:**
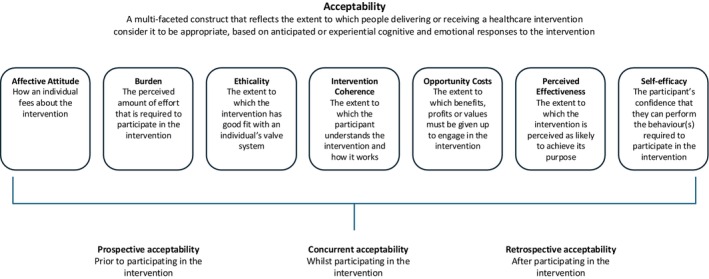
Sekhon's original Theoretical Framework of Acceptability.

For this study, the framework provides a rigorous theoretical lens from which to interpret multiple contributors to the perceived acceptability of TAVI across three temporal zones: prospective (attitudes toward the intervention beforehand), concurrent (experience while having the intervention) and retrospective (reflecting on the experience of the intervention). The seven domains of interpretation may explain acceptability and the varying extents of influence across each temporal zone (see Figure 1).

The seven domains were re‐ordered to match the patient participant journey and to organise a description of the acceptability of TAVI for patient and carer participants, by exploring:

1. How the patient participants seemed to construct their identity and to use this as a backdrop to understand their prospective response and attitude to AS and TAVI (ethicality, affective attitude and intervention coherence)
2. The TAVI procedure itself (concurrent acceptability) and how this impacted patients' lives (opportunity costs, burden)
3. The patient's ability to recover and return to a normal life post‐operatively—retrospective acceptability—(perceived effectiveness, self‐efficacy)

**FIGURE 2:**
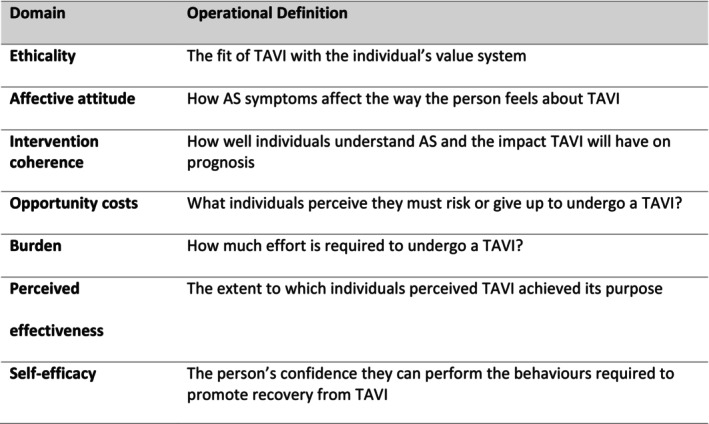
Theoretical Framework of Acceptability domains: Operational definitions of each construct.

Within each of the seven acceptability domains, further subthemes were developed inductively to validate the framework for application to the TAVI procedure. As the Acceptability Framework was developed by Sekhon for a different intervention, we needed to clarify what these theoretical domains meant for our study, prior to analysis. This approach is an important element of directed content analysis (Hsieh and Shannon 2005) and has been described in other acceptability studies (Sheils et al. 2020) (see Figure 2).

Acceptability is a key component of implementation science as it reflects how those delivering or receiving a healthcare intervention perceive it as appropriate, reasonable or satisfactory. This study applies Sekhon's Theoretical Framework of Acceptability to explore how older patients and their carers perceive TAVI, offering insights into barriers and facilitators to its real‐world integration. By incorporating patient and carer perspectives, the study contributes to implementation literature with strategies to enhance engagement, recovery and support for future TAVI patients.

### 3.3 Study Setting and Recruitment

Patient participants were recruited from three metropolitan hospitals in Australia between 2018 and 2021. Patient participants were eligible if they were aged ≥ 18 years, able to provide written informed consent, and had undergone the TAVI procedure in the last 4–6 months. Carers were identified by an enrolled patient participant as a person who provided them with close care and support. Participants were approached by a research team member who did not provide direct clinical care. Study information and consent were provided to potential participants (both verbally and written) to review and consider enrolment in their own time, and participants provided written informed consent. Initially, purposive sampling, seeking maximum variation in age, gender and carer relationship, was employed. Thereafter, theoretical sampling refined participant selection to provide insight into underdeveloped aspects of the emerging deductive analysis.

### 3.4 Data Collection

An interview guide developed collaboratively by the research team (all experienced in the care of chronic heart conditions) was informed by the theoretical domains of acceptability and relevant literature. This initial version of the interview guide was peer‐reviewed by clinicians and qualitative research experts. Minor amendments were made for clarity and coherence, then refined following a staged process (Kallio et al. 2016). Demographic data collected included the patient's age, sex, health conditions and living situation (geographical location and cohabitation status).

Participants engaged in a single semi‐structured interview between 2018 and 2021. Interviews were scheduled at a mutually convenient time, by telephone or face‐to‐face in the recruiting hospitals in a location separate to routine care delivery (e.g., private offices) (Table 1). The interviewer (NS) was trained in qualitative interview techniques, and median interview duration was 37 min. Interviews were audio‐recorded and transcribed verbatim.

**TABLE 1:** Participant characteristics.

Patient pseudonym, (age years) and marital status	Carer pseudonym, relationship to patient status	Patient health conditions concurrent to AS	Patient geographical location and cohabitation status
Paula [75] De‐facto	Trisha Partner	Chronic cardiovascular and renal conditions. Active, impacting condition: anaemia.	Metropolitan, assisted living complex with partner.
Mike [83] Married	Susan Wife	Chronic cardiovascular condition. Active, impacting conditions: rheumatism, insomnia.	Regional, lives with wife.
Martin [82] Married	Linda Wife	Active impacting condition: falls	Metropolitan, lives with wife.
Bridget [84] Married	Tracey Daughter	Active impacting condition: anxiety.	Regional, lives with husband.
Roy [82] Married	Mary Wife	Chronic cardiovascular condition. Active impacting condition: falls.	Metropolitan, lives with wife.
Richard [93] Widowed	Jack Son	Active impacting conditions: musculoskeletal pain, arthritis, ulcerative colitis.	Metropolitan, lives alone.
Trevor [84] Married	Angela Wife	Chronic cardiovascular and neurological conditions.	Regional, lives with wife.
Greg [80] Married	Maureen Wife	Active impacting conditions: musculoskeletal pain, falls.	Regional, lives with wife.
Anne [99] Widowed	—	Chronic cardiovascular, respiratory, digestive conditions. Active, impacting conditions: falls.	Metropolitan, lives alone.
Donald [77] Married	—	Chronic cardiovascular, respiratory, renal and endocrine conditions. Active, impacting conditions: musculoskeletal pain, leg ulcers.	Regional, lives with wife and daughter.
Victor [89] Widowed	—	Chronic cardiovascular, respiratory, renal and endocrine conditions. Active, impacting condition: foot ulcer.	Regional, lives alone.
Len [77] Married	—	Chronic cardiovascular and endocrine conditions. Active, impacting condition: anaemia.	Regional, lives with wife.
Mary [94] Married	—	Chronic cardiovascular and renal conditions.	Metropolitan, assisted living with husband.
Bruce [87] Widowed	—	Chronic respiratory condition. Active impacting conditions: deaf, musculoskeletal pain.	Regional, lives alone.
Cathy [79] Widowed	—	Chronic cardiovascular condition.	Metropolitan, retirement village lives alone.
Ursula [88] Widowed	—	Chronic renal condition.	Metropolitan, lives alone.
Derek [85] Married	—	Chronic cardiovascular condition. Active impacting condition: poor eyesight.	Regional, lives with wife.
Duncan [87] Widowed	—	Chronic renal condition. Active impacting condition: anaemia.	Regional, lives alone.

*Note:* —, no carer interview.

Sampling occurred in two stages. First, purposive sampling was employed to capture a broad range of participant characteristics—such as age, gender, and carer relationship—to ensure diverse perspectives (*n* = 16). This was followed by theoretical sampling to recruit participants (*n* = 10) who could provide deeper insight into underexplored areas—such as intervention coherence and self‐efficacy—identified during the ongoing deductive analysis. Sample size was guided by the richness (a multilayered quality) and thickness (quantity) of data (Malterud et al. 2016). Recruitment continued until major themes were richly developed, including negative case examples (Fugard and Potts 2015). Interview transcripts were deidentified, and the transcribed text was checked against the interview audio file by the interviewer.

### 3.5 Data Analysis

A directed content analysis approach was used to examine the data, guided by the Theoretical Framework of Acceptability (TFA) (Sekhon et al. 2017). This approach allowed for deductive coding of the interview transcripts into the seven predefined TFA domains: affective attitude, burden, ethicality, intervention coherence, opportunity costs, perceived effectiveness and self‐efficacy. Directed content analysis is particularly suited to validating or extending an existing theoretical framework within a new population or context (Hsieh and Shannon 2005), making it appropriate for exploring the acceptability of TAVI in individuals with AS and their carers.

The analytic process involved multiple steps. First, interview transcripts were read in full to achieve familiarisation. Initial coding was then undertaken by mapping participants' responses to the relevant TFA domains. Text segments were coded deductively according to domain definitions, while allowing for emergent content within each domain. Where data did not clearly align with the predefined categories, codes were discussed among the research team and either reinterpreted or retained as potential extensions of the original framework. To ensure trustworthiness, coding consistency was checked by multiple team members, and discrepancies were resolved through discussion.

Negative case analysis was also incorporated to identify data that contradicted dominant themes. These cases were intentionally sought to challenge assumptions and ensure a nuanced and balanced interpretation. While most participants reported high acceptability of TAVI, negative cases highlighted that acceptability can vary depending on individual health status, expectations and life context.

**FIGURE 3:**
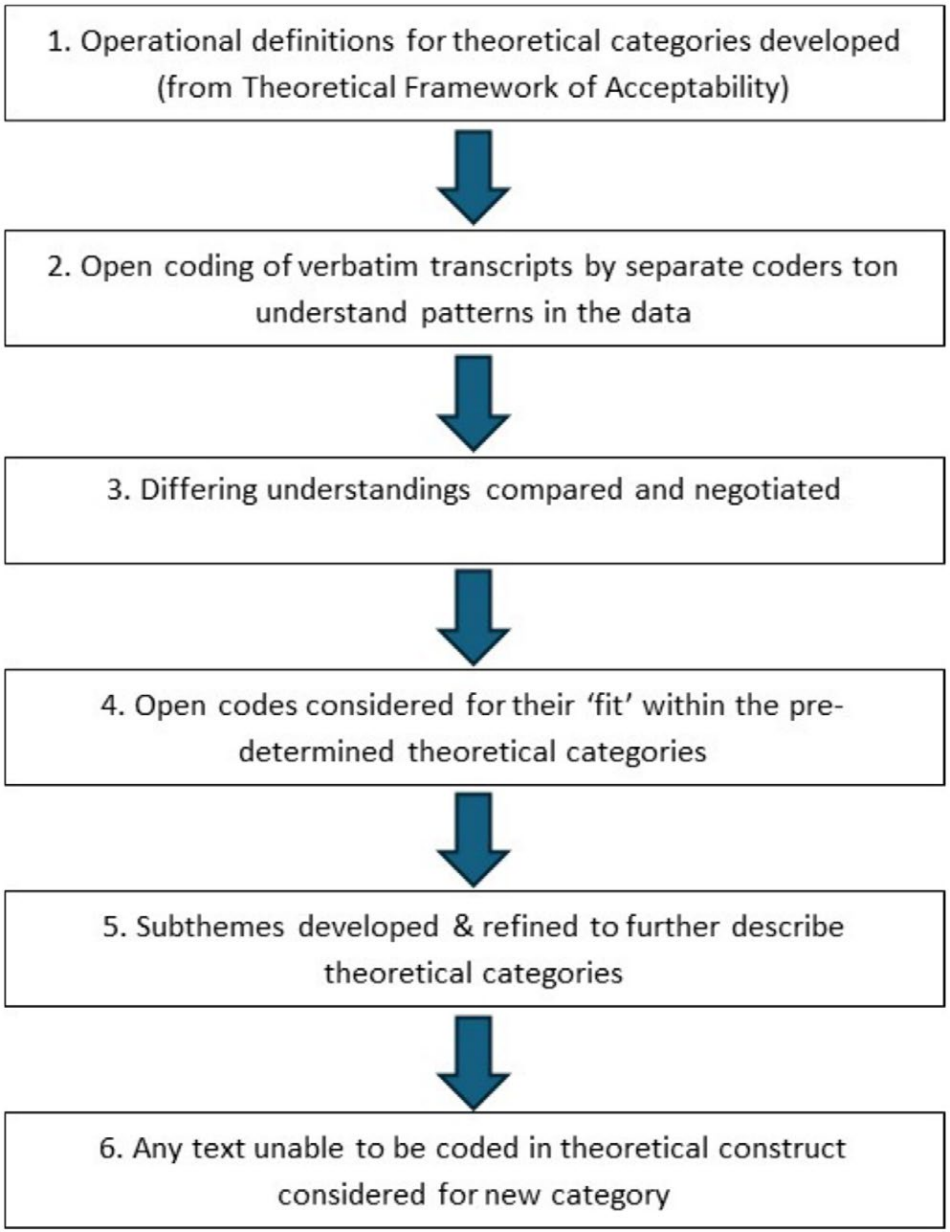
Thematic coding and data analysis process.

A visual summary of the analytic process is presented in Figure 3.

### 3.6 Rigour

The *credibility* of the study design was supported by utilising Elo et al.'s Checklist for Trustworthiness in Qualitative Content Analysis (see File S1) (Elo et al. 2014). This manuscript was also prepared in accordance with the Consolidated Criteria for Reporting Qualitative Research (COREQ) checklist (Tong et al. 2007) (File S2). *Conformability* was supported by two researchers (NS and JG) independently reviewing the transcripts and collaborating on the analysis, which was iterative in nature. Data analysis and resolution of discrepancies or challenges were guided by a consensus approach involving two primary researchers and a third reviewer (RG), who critically challenged assumptions and contributed to refining the interpretation of findings and improving the coherence of our reporting. This third researcher later reviewed the analysis process and findings with ‘fresh eyes’, using the raw transcript data as a reference point to confirm accuracy. *Transferability* was supported by a detailed presentation of the data, sampling and participant characteristics, and the multi‐site design (Elo et al. 2014).

### 3.7 Ethical Considerations

Written informed consent was obtained from all participants. The informants' identities were protected by avoiding detailed descriptions of unique situations and by deidentifying participants in transcripts and reports through the use of pseudonyms. The Northern Sydney Local Health District Human Research Ethics Committee (Ref: RNSH ETH00671) approved the study protocol. A participant distress protocol was in place but was not required.

## 4 Results

The sample of 26 participants included 18 patients: 67% male (*n* = 12) and 33% female (*n* = 6) and 8 carers: 75% partners (*n* = 6) and 25% adult children (*n* = 2) (see Table 1). Patient participants had a mean age of 84.2 ± 4.1 years; 67% (*n* = 12) lived with a family member, 33% (*n* = 6) lived alone and 39% (*n* = 7) reported more than 5 comorbidities, most commonly cardiovascular, respiratory and renal disease.

**FIGURE 4:**
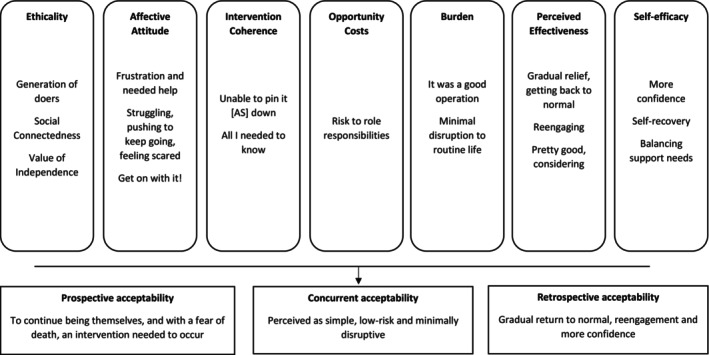
Superimposing the perspectives and experiences of patients (and their carers) with AS undergoing TAVI into the framework of acceptability.

The seven domains of the Theoretical Framework of Acceptability structured the initial deductive analysis. To deepen understanding, inductive thematic analysis was conducted within each domain to identify sub‐themes that reflected the contextual nuances of participants' experiences. This approach allowed for a more detailed exploration of how patients and carers understood and evaluated TAVI in relation to their individual circumstances (see Figure 4).

### 4.1 Ethicality

#### 4.1.1 Generation of Doers, Social Connectedness and Value of Independence

To understand the ‘fit’ of TAVI within an individual's value system (ethicality) it was important to examine how these patient participants constructed their identities to understand their perceptions of self, their ethical values, and the subsequent impact on their experience of the new AS diagnosis, symptom management, treatment decisions and post‐operative recovery. Most patient participants were born between 1930 and 1940 and seemed to identify as a *generation of doers* who adapted to meet life course demands and valued *social connectedness*. They constructed their identities around their contributions to family, friends and communities, and placed a strong emphasis on maintaining and *valuing independence*.

The oldest patient Anne (99 years) discussed her *ethicality* in the context of her early career path, given the contributions available to women of her generation, explaining: ‘I did chemistry and was a laboratory assistant for 10 years… but in those days, you had to stop working when you got married’. Carers recognised and benefitted from the altruism demonstrated by this *generation of doers*. Tracey (Daughter of Bridget, 84 years) discussed how she valued her mother's sense of ethicality as her own parental responsibilities increased, saying ‘She was great when my kids were little, a lot of childcare’. Whereas for others a strong sense of *ethicality* stemming from a *generation of doers* enabled some patient participants to pursue multiple career paths over the years. Derek's (85 years) explanation demonstrated agility in his approach to work life decisions; ‘I started off in the bank for 22 years, then the pub business, then I bought a cab and then I decided to buy a health foods shop…then I went to university, and I came out with an MA in Latin, then got a job at the college until I retired for the second time’.

Maintaining *social connectedness* to family, friends and community was also important to patient participants. Just over half reported their family members living nearby, and others maintained regular contact with family despite geographical distance. Some expressed appreciation for connectedness with friends and community members who provided emotional and practical support. Bruce (patient, 87 years) said of his friend of 40 years, ‘he comes down one day a week and does anything that I want done or fixed, and he takes me shopping’. Several participants lamented a decline in their social connectedness as their friends grew older and experienced health challenges.

As the symptoms of AS emerged and increased, the ethical value of *social connectedness* expressed by the *generation of doers* was challenged as many reduced their visits with extended family and friends before the procedure, due to the physical effort and energy required. Mike (83 years) described how his increasing dyspnoea decreased social connectedness: ‘The trips to [son's home] became more rare… and often my [wife] would go on her own’. This trade‐off was needed by patients to maintain their functional reserve and preserve their energy for basic demands and activities of daily living. The reduction in independence led to a greater reliance on others. This posed a further challenge to the ethical values of this *generation of doers* who strongly *valued their independence*, the autonomy to make their own decisions, and their capacity to live in, and be an active part of, their surrounding environments and communities.

### 4.2 Affective Attitude

#### 4.2.1 Frustration and Needed Help

The *affective attitude* and subsequent *prospective acceptability* of TAVI for many patient participants was directly associated with worsening symptoms, such as increasing shortness of breath and fatigue. Prospective acceptability was attributed to a growing inability to do *the little things* that had previously taken for granted, resulting in an *affective attitude* of *frustration*, especially when they *needed help* from others. About a third of patient participants shared this frustration and had to rethink and adapt to manage the most basic daily tasks that were easy to perform before AS. Paula (75 years) who lived with her partner, shared how this challenged the independence she so deeply valued: ‘If I wanted to make myself a cup of coffee or tea, it would take me three goes. One to put the kettle on, one to sit down for five, and one to make the pot…so frustrating… I hated it’.

Anne (99 years) who lived alone, also expressed how not being able to do *the little things* led to her affective attitude of *frustration* and perhaps in turn, a prospective acceptability of TAVI:

‘It was infuriating, because there were things that I used to do so easily… little minor things, opening a can, that sort of thing that require a little bit of effort… little things, but things that rule the way you live your life’.

Martin (82 years) recalled his realisation that he *needed help* to maintain independence: ‘the frustrations we had was getting to [doctors] appointments’. Whilst many patient participants described *needing help*, some admitted that they did not seek help. Reasons for not seeking help perhaps aligned with the ethicality of a *generation of doers* and their *value of independence* and may have positively influenced the prospective acceptability of TAVI.

#### 4.2.2 Struggling, Pushing to Keep Going, Feeling Scared

Most patient participants adapted their lifestyle to cope with AS, and while this was made easier with social support, the symptom burden left them *struggling* and *feeling scared*: affective attitudes likely to influence the prospective acceptability of TAVI. Richard (93 years) revealed how badly he was *struggling* and what this meant for his ability to perform basic activities of living, ‘I was suffering very badly from breathlessness and pain…I couldn't walk from one room to another without having to sit down’. Paula (75 years) explained: ‘I was literally struggling to breath, I thought I was nearly gone… very scary’. Carers also noticed this dramatic symptom burden, the *struggling* and the negative impact on basic tasks. Jack (son of Richard, 93 years) explained: ‘extremely out of breath with the least exertion. He'd get into the shower and then halfway through showering himself, he was gasping for breath’.

Nevertheless, being from a *generation of doers*, patient participants living alone or with caring responsibilities were unwilling to let their AS symptoms stop their responses to life's demands. Bridget (patient, 84 years and carer for her husband with dementia) said: ‘I was looking after my husband, my home, the shopping … and I was getting more breathless and tired. I was pushing myself at times to keep going’.

#### 4.2.3 Get on With It!

Whilst not all patient participants experienced severe symptoms, nearly all expressed a desire to *get on with it* [TAVI]. There was a growing sense of treatment urgency that defined the *affective attitude* for participants who were *struggling* and *pushing to keep going*, confirming the *prospective acceptability* of TAVI. This attitude seemed to be influenced by their ethical values as a *generation of doers*, where identifying a problem led to a need for an immediate solution or action: Victor (89 years) said post‐diagnosis: ‘Well, if it's bloody fixable, fix it!’. Similarly, carers also conveyed an affective attitude of ‘get on with it’. For Tracey (daughter of Bridget, 84 years), this may have arisen from feelings of responsibility, helplessness and uncertainty: ‘I was sort of on standby to go and be with her when she had the operation. It was all dangling a bit’.

### 4.3 Intervention Coherence

#### 4.3.1 Unable to Pin It [AS] Down and All I Needed to Know

Intervention coherence described the extent to which patient and carer participants understood AS, its prognosis, and the expected outcomes before, during and after TAVI. Inductively derived themes included being *unable to pin it down* (a lack of a clear understanding of the biological cause of AS, and associated symptoms) and having *all I needed to know* (enough information requirements about AS and TAVI pre‐operatively, yet inadequate information about operative success).

The ambiguity of being *unable to pin it down* sometimes related to which current health issue was causing symptoms of breathlessness and fatigue. This resulted in an initially low intervention coherence. In particular, carers had difficulty making this distinction and suspected symptoms originated from existing co‐morbidities. Anne (99 years) describes her initial lack of intervention coherence arising from age‐related decline and her health literacy: ‘I thought that I was getting pretty old…I had no idea what part the valve played in your living… you just think the heart is a little red ball’. Being *unable to pin it down*, meant many patients were surprised once the diagnosis of AS was confirmed. Progressive sense‐making behaviours, to enable *intervention coherence*, often started with the emergence of symptoms, Mary (94 years) recalled: ‘I couldn't believe it when I went to the doctor that morning, and I only went because I didn't have the same energy as I normally have’.

Eventually, most patient participants could distinguish between symptoms of worsening AS and other existing health conditions. Some participants described additional information‐seeking behaviours to understand AS and corroborate medical advice, while others were content to be informed by treating clinicians. Two carers described how information about risk arising from informed consent may have negatively influenced intervention coherence and prospective acceptability of TAVI. Tracey (daughter of Bridget, 84 years) explained that ‘some of [mother's] anxiety was related to the way she interpreted the information that she got’. Trisha (partner of Paula, 75 years) described the fine balance between addressing the uncertainty of being *unable to pin it down*, versus the personalisation of information to support intervention coherence and prospective acceptability of TAVI: ‘It's nice to ensure that people are fully informed, but increasingly we see the risk management system bombarding people with information, so they don't really get what is important in it’. Regardless of the variations in understanding of AS and TAVI, most patient participants said that pre‐operatively, they had *all they needed to know*, with their descriptions revealing a high prospective and concurrent acceptability of TAVI for the domain of intervention coherence.

Whilst TAVI may have been a clinical success, carers particularly were unsure of how the valve structure and function had changed, nor what this meant for long‐term prognosis. Mary (wife of Roy, 82 years) highlighted how a lack of peri‐operative information left her feeling anxious: ‘They could have been more informative of what they did… I'd have felt more relaxed in knowing what to do and what had happened to him. But they didn't even tell me whether the operation went well or not. There was no communication’. Retrospective acceptability was, subsequently, negatively impacted as carers highlighted feelings of apprehension about discharge and a lack of awareness about post‐operative responsibilities and expectations. Tracey (daughter of Bridget, 84 years) stated, ‘It was sort of like, “Yeah, off you go”’.

In the early post‐discharge and recovery phase, Trisha (partner of Paula, 75 years) explained that neither of them had *all they needed to know* about post discharge recovery, led to an affective attitude of fear and uncertainty about physical limits. This likely challenged intervention coherence and influence their retrospective acceptability of TAVI: ‘I think it would help…because she was… and still is, a bit fearful of overdoing it and giving herself a setback’.

### 4.4 Opportunity Costs

#### 4.4.1 Risk to Role Opportunities

For all but one participant, it appeared that the opportunity costs to undergo a TAVI were low (i.e., extent to which benefits, profits or values might be negatively impacted by engaging with the intervention).

One patient participant did articulate a potential consequence, being a ‘risk to role responsibilities’ (the threat of being unable to perform role obligations). For Bridget (TAVI patient, 84 years), the pre‐operative decision‐making process incorporated her obligation as full‐time carer for her husband, who's dementia‐related support needs were increasing: ‘They explained both the operation… and the consequences, and my biggest worry was… I knew that my husband needed more help than I had realized’. With only one participant describing a possible *opportunity cost*, this supports the *prospective acceptability* of TAVI to participants, and their desire to just *get on with it*.

### 4.5 Burden

#### 4.5.1 It Was a Good Operation

Considering the older age and multiple comorbidities of most patient participants, intervention burden is an important factor in determining the acceptability of TAVI, with carers expressing concerns about the potential risks and benefits of the intervention for their loved ones. The first theme in the burden domain was that *it [TAVI] was a good operation*, having small incisions, minimal pain and straightforward recovery within the first 24 post‐operative hours, resulting in high concurrent acceptability. Mike (83 years) said ‘it was a good operation, skilfully done, and I came out from the operation feeling mentally a bit more sparkly’. Derek (85 years) explained: ‘it was over in 20 minutes…completely painless’. Carers concurred with this perspective, with both cohorts measuring burden against actual physical impact to the body or previous clinical procedures.

Despite the mainly positive perceptions of intervention burden, intraoperative healthcare delivery events impacted concurrent acceptability for two patient participants. Bruce (87 years) experienced a temporary vascular complication, while Duncan (87 years) described the burden of the intervention amid the COVID‐19 pandemic, which created uncertainty around his recovery.

#### 4.5.2 Minimal Disruption to Routine Life

Secondly, the *minimal disruption to routine life* by both patients and carers was related to relatively short hospital stays of 3–5 days, again leading to high concurrent acceptability of TAVI. Donald (patient, 77 years), referencing a previous cardiac operation, said: ‘after I had my bypasses done, I seemed to be worse… TAVI wasn't much to me, they operated on the Wednesday morning, and I was in my hotel on Friday afternoon. I was good’.

Carers also welcomed the minimally disruptive experience: Susan (wife of Mike, 83 years) noted ‘It was a very quick [intraoperative] recovery’; and Jack (son of Richard, 93 years) said ‘it was only a few days… I don't think he was in there for a week’.

### 4.6 Perceived Effectiveness

The perceived effectiveness of TAVI is crucial in understanding the retrospective acceptability of the intervention among patients and carers, as the primary goal of TAVI is to treat severe symptomatic AS and improve mortality, morbidity and quality of life outcomes. Three prominent themes emerged to explain the perceived effectiveness of TAVI, including *gradual relief and getting back to normal*, *reengaging* with people and pastimes, and feeling *pretty good, considering*.

#### 4.6.1 Gradual Relief, Getting Back to Normal and Reengaging

For over half the participants', positive perceived effectiveness was connected to improvement in physical health (*a gradual relief* and *getting back to normal*) and mental wellbeing (*affective attitude*) post TAVI. Anne (99 years) explained: ‘I was expecting a sort of miraculous change. That didn't happen… the first week after it, I thought ‘Well, that doesn't feel any different.’ Then gradually, I suddenly realized, “Oh gosh, I am not panting!”.’

Paula (75 years) expressed a regaining of important elements of personal lived meaning, and this improved her *affective attitude* of struggling: ‘I have gained a lot of my life back, because… I'm not struggling’. Len (77 years) described his improved affective attitude supporting perceived *effectiveness* and *retrospective acceptability* post‐TAVI: ‘I have seen a remarkable change since the TAVI…because I now can relax more’. Carers also confirmed retrospective acceptability. Jack (son of Richard, 93 years) conveyed his father's perceived effectiveness: ‘He said it was a life‐changing event and he can breathe really good again’. Tracey (daughter of Bridget, 84 years) expanded as she described her mother's behaviour and motivation to engage with, and participate in, a cardiac rehabilitation programme post‐operatively: ‘She was laughing and joking… she's got a lovely sense of humour and I really saw that coming out. She was optimistic in a way that I hadn't seen for a while’.

Despite perceived effectiveness being commonly described as *a gradual relief* in AS symptom burden, at 4–6 months post operatively, more than half expressed some mild ongoing symptoms of breathlessness and fatigue. Several patients and carers reflected that overall, their general health had not improved significantly, and this could be linked to other chronic health conditions, overall deconditioning or advancing age. Richard (93 years) described how comorbid arthritis challenged his function even though AS symptoms improved: ‘I got to use the walking frame and I can't do any shopping. I can't do anything. I'm just confined to the house… I've got no life, really’.

Mary (wife of Roy, 82 years) also reflected on the variability of her husband's health saying: ‘he has good days and not‐so‐good days. He did improve, but I guess age is against him… I think we've got to be sensible and realise that doctors are good, but they can only do so much’. The moving goalposts of ‘normal’ was confronting when patient and carers' expectations (and therefore, the retrospective acceptability of TAVI) were not fully met. Roy (82 years) expanded saying: ‘I was expecting a miracle. It's rough because I'm not doing what I should or what I thought I could do. I'm just thinking maybe I expected too much’.

#### 4.6.2 Pretty Good, Considering

Regardless, most patients and carers described their overall general health as *pretty good considering*. Given these patients were often managing multiple co‐morbidities, and through their ethicality lens, AS was just another condition that had to be dealt with. This may have favourably supported perceived effectiveness and retrospective acceptability at 4–6 months post‐TAVI.

### 4.7 Self‐Efficacy

The operational definition of self‐efficacy was adapted to focus on the person's confidence that they could perform the behaviours required to promote recovery from TAVI and was analysed retrospectively based on the acceptability of the intervention. The themes of *more confidence*, *self‐led recovery*, and *balancing support needs* were identified to explain participants' self‐efficacy.

#### 4.7.1 More Confidence and Self‐Led Recovery

Most patient participants had *more confidence* to reengage with their normal lives and activities post‐TAVI, which in turn led to improvements in mental wellbeing, and this was also noted by carers. Paula (75 years) explained, ‘I'm gaining confidence each week. It's such a sense of freedom…to be able to just do as I used to do’. Most patient participants (17 out of 18), described a *self‐led recovery*, planning and initiating their own physical rehabilitation. Martin (82 years) described: ‘some brisk walking…and on the alternate days, my wife has one of these pedalling machines and I get on that for a half‐an‐hour’.

The first reason for this *self‐led recovery*, was a lack of structured discharge advice about recovery and rehabilitation. Roy (82 years) said ‘…all they said was ‘go home and take it easy’ and for the TAVI to settle in and get used to what I'm doing’. The reason for a *self‐led recovery* related to the geographical availability and/or accessibility of healthcare services. Derek (85 years) described how the lack of cardiac rehabilitation programmes in his area negatively impacted his physical recovery and that he relied upon his self‐efficacy to improve his physical ability post‐TAVI: ‘The local hospital isn't there anymore… and I don't drive so… I rested for about 6‐weeks… I got walking, not very far at first… very slowly and it gradually improved… by another 6‐weeks I was back to walking normally’.

Bruce (87 years) highlighted his difficulty and confusion in interpreting recovery guidance from inconsistent resources (e.g., both open‐heart surgery and percutaneous procedure leaflets), alongside his inability to apply the advice to his own environment and life stage:

‘I've got two books [given to me]. One book… had the open‐heart program in it, and the little book with the groin one [transluminal procedures like TAVI]… It tells you what I should be doing… I should be walking a mile every day, but I can't… because I'm up on the hill and the road down in front is a little bit narrow’.

#### 4.7.2 Balancing Support Needs

As these older persons recovered and reengaged with their normal lives, they re‐evaluated the social support needs required to maintain independent living. The majority of patient participants were able to *balance their support needs* effectively, enabling them to live independently. Carers' perspectives did not always align, suggesting support did not always balance with needs, and that more assistance might be required now or in the near future.

## 5 Discussion

Sekhon et al.'s acceptability framework offers a comprehensive theoretical structure that facilitated the analysis of both shared and unique experiences, revealing subtle yet significant distinctions in how individuals perceive illness and treatments. In this study, patients and carers consistently viewed TAVI as a highly acceptable intervention for aortic stenosis, largely due to its role in relieving symptoms and enabling a return to everyday life. High acceptability was driven by improvements in physical and mental well‐being, the perceived effectiveness and low burden of the procedure, and increased self‐efficacy that supported re‐engagement in valued activities.

Our findings support earlier and more recent evidence that the AS condition challenges patient's coping experiences whilst awaiting AVR treatment (Berg et al. 2013; Helder et al. 2019; Skaar et al. 2017). In one study, people with AS described ‘living on the edge but trying to stay in control’; conveying the daily struggle and negative impact of AS on physical and mental well‐being (Olsson et al. 2016). This study adds to these findings by including the missing voice of carers, who provide the bulk of support across the AS‐TAVI continuum. Carers confirmed the debilitating symptomatic experiences of people with AS, but also expressed their own emotional distress during the waiting period before TAVI. Similar experiences have been described by carers of people with heart failure, who are often unsure how to cope with the symptom variability, what to do in an emergency or how best to provide emotional support during the fluctuating and worsening health of their loved ones (Pattenden et al. 2007; Wingham et al. 2015). For the carers in this current sample, the high prospective acceptability of TAVI was thus influenced by a combination of seeing the people they cared about deteriorate due to increasing AS symptoms, and their own experiences of uncertainty and anxiety.

People with AS perceived they had little to give up to undergo TAVI, and potentially much to gain. Earlier studies provide similar insights into AS patient's treatment hopes and goals as they await TAVI, finding many expect to ‘get back to normal’, ‘reverse the tide of increasing fatigue’ and ‘reconnect with their social network’ (Astin et al. 2017; Lauck et al. 2016). Only one patient and her carer (daughter) in our study identified the perceived threat of being unable to perform role obligations whilst undergoing, and/or recovering from TAVI. This participant was the main carer for her husband living with dementia in their own home, revealing the importance of pre‐operative psychosocial assessment for discharge planning, as diverse roles performed by many older people are often overlooked. While the decision between TAVI, surgery or conservative management should ideally consider clinical factors, technical suitability and patient preferences, treatment choices are typically made by heart teams. As a result, shared decision‐making, which takes into account patients and carers perspectives is often underutilised, despite its importance in enabling person‐centred care (Montori et al. 2023).

Any negative pre‐procedure emotions about TAVI directly contrasted with the post‐operative positivity expressed by AS patients and confirmed by carers. Post‐TAVI, participants described being able to breathe again, to do as they used to do, gaining a sense of freedom, and having the ability to relax. Our findings also demonstrate that before AVR, people with severe AS often experience debilitating symptoms that lead to dependence on others. However, after TAVI, most experienced a return of functional independence to at least pre‐AS levels. This resonates with recent findings that post‐TAVI, patients experienced greater trust in their own bodies and an ability to resume everyday activities (Kirk et al. 2019).

Expectations for symptom relief were realistic among the patients and carers; however, return to function was much slower than expected. Most patient participants found it took longer than anticipated to increase their walking or to perform their routine activities of living with ease. Expectations for symptom relief were generally realistic among patients and carers; however, many found that returning to functional independence—such as walking and performing daily activities—took longer than expected. This contrasts with findings from a large study showing more rapid improvements in function and symptom burden after TAVI compared to surgical valve replacement (Tuttle et al. 2022). Notably, participants in that study were typically younger (mean age 79.8 ± 6.2 years) and at intermediate surgical risk, which may partly explain the discrepancy.

These differences highlight the importance of considering patient‐specific factors when evaluating the acceptability of TAVI. Acceptability is not universal—what is deemed acceptable by one patient group may not be by another. Factors such as older age, lower baseline health status, non‐transfemoral access and limited social support have been linked to poorer quality of life outcomes at 30 days post‐procedure (Lauck et al. 2021). This underscores the need for personalised assessments that account for both clinical and contextual factors when considering TAVI acceptability by patients and carers.

Research exploring physical activity after myocardial infarction also described people accepting a ‘new normal’ (Birtwistle et al. 2022). This complements other qualitative literature on AS and TAVI with diverse recovery experiences reported (Lysell and Wolf 2021; Olsson et al. 2018). Both previous work and ours provide deeper insights into exactly what recovering at one's own pace and in one's own environment means for AS patients and their carers. This underscores the pressing need for detailed physical and psycho‐social assessment and individualised support to optimise the rehabilitation process.

Our work contributes to the expanding field of healthcare intervention acceptability research. It demonstrates the usefulness and transferability of Sekhon et al.'s Acceptability Framework in understanding high‐risk, high‐benefit interventions within an older patient population, as demonstrated in other complex clinical procedures and programmes (Jones et al. 2021; Ndejjo et al. 2020).

Applying the Theoretical Framework of Acceptability provides a structured foundation for designing and delivering more effective, person‐centred healthcare interventions. The framework underscores the pivotal role of nurses, who are deeply embedded in the patient care journey. Nurses naturally embody the principle of ethicality, trained to value and support the whole person (Ward and Swanson 2020). Their communication skills are crucial—translating complex medical information into plain language and facilitating shared decision‐making by exploring a person's illness and treatment concerns alongside their goals and values (Pan and Wang 2025). In TAVI this was demonstrated through the nurse's role in education and pre‐ and post‐procedural care. Through clinical practice and research, nurses play a key role in shaping evidence and care that is aligned with the needs, preferences and lived experiences of patients with aortic stenosis.

## 6 Strengths and Limitations

This study has several strengths and limitations. Sekhon et al.'s Theoretical Framework of Acceptability provided a structured and rigorous analytic scaffold for the data and allowed examination of the specific theoretical domains that lead to TAVI being a highly acceptable intervention to both AS patients and their carers (Sekhon et al. 2017). These findings may be transferable to similar surgical contexts and patient cohorts. Participants were recruited across three hospitals, enabling a diversity of voices and perspectives to be represented.

A limitation of this study is the data collection period and the age of the data, which was affected by two external factors beyond the researchers' control. Initially, from 2018 to 2021, TAVI had only recently been added to the Australian Medicare Benefits Schedule (November 2017), and participating hospitals were performing just 20–30 procedures annually, limiting the pool of eligible participants. As procedure volumes began to increase, the onset of the COVID‐19 pandemic further disrupted TAVI services and required protocol amendments, including additional ethics approval to allow interviews to be conducted by phone as well as in person. Another potential limitation was the imbalance between patient and carer participants. Many patients were widowed, and some potential carers were either elderly or lived too far from the study site to participate. Nonetheless, including patients without carer support provided valuable insights. All participants were also English‐speaking, which may limit representation of Australia's culturally and linguistically diverse population. Additionally, as interviews were conducted 4–6 months after TAVI, there is a possibility of recall bias affecting the accuracy of participants' reflections of their experiences. Furthermore, as this was a qualitative study with a relatively small and context‐specific sample, the findings are not intended to be generalisable to the broader population of patients who undergo TAVI. Future research could address this by involving patients and carers in study co‐design and using interpreter services to ensure broader inclusion.

## 7 Conclusion

This study confirmed TAVI as a highly acceptable intervention for both patients and their carers. TAVI reduced the emotional toll of struggling with symptoms, fear and frustration, and the procedure was perceived as having low pre‐operative risk and intraoperative burden, which eased concerns about potential complications to both patients and carers. In addition, and acknowledging this cohort of older AS patients, TAVI aligns with the ethical values of this *generation of doers* who prioritise minimally invasive interventions that allow them to maintain their independence, contribution and quality of life.

## 8 Implications for Policy and Practice

Robust, systematic exploration of AS patient and carer perspectives about intervention acceptability provides a better understanding of consumer preferences, values and expectations about TAVI. While there was some variability in experience, understanding the generally high acceptability of TAVI enables better patient/carer education and decision/rehabilitation support for the mostly elderly patients and their carers, especially in the context of same‐day discharge. The establishment of multidisciplinary communication pathways is being explored, yielding positive early results in terms of safety and effectiveness. There is further potential to expand nurses' scope of practice, fostering an understanding of the importance of shared decision‐making in AS, both before and after TAVI and how to embed these processes into clinical practice. This approach is not only person centred; it also has potential to be cost‐effective and sustainable.

A safe and well‐supported discharge process for TAVI patients, with or without the aid of carers, requires a clear and comprehensive discharge plan that should begin in the pre‐operative period and incorporate tailored recommendations for recovery and rehabilitation based on individual physical constraints and environmental factors. Ongoing follow‐up and prognostic considerations could inform appropriate emotional support embedded throughout the continuum of care.

## Author Contributions

All researchers conceptualised and designed the study. N.S. collected data. N.S. and J.G. analysed data, with R.G. providing critical comment on analysis and all authors interpreting the emerging findings. N.S. drafted the manuscript. All authors provided critical revision and approved the final version.

## Conflicts of Interest

The authors declare no conflicts of interest.

## Supporting information


**Table S1:** jan70180‐sup‐0001‐TableS1.docx.

**Table S2:** jan70180‐sup‐0002‐TableS2.docx.

## Data Availability

The data are not publicly available since allowances for public data sharing were not included in the informed consent procedures.
