# Supplementary material for: A Descriptive Qualitative Study of Patient and Carer Perspectives on the Acceptability of Transcatheter Aortic Valve Implantation
Source: J Adv Nurs. 2025 Sep 3;82(5):5305–18. doi: 10.1111/jan.70180 (PMC13069208; doi:10.1111/jan.70180)
Supplement: Supplementary file 1 — Table S1: jan70180‐sup‐0001‐TableS1.docx. [file JAN-82-5305-s001.docx]

Supplementary file 1

# Appendix B: Checklist for trustworthiness in qualitative content analysis

| **Phase of the content analysis study** | **Questions to Check** | **Responses** |
| --- | --- | --- |
| **Preparation phase** | | |
| Data collection method | How do I collect the most suitable data for my content analysis? | individual conversational-style interviews with patients and their carers. |
|  | Is this method the best available to answer the target research question? | Yes, to explore central perspectives of the people undergoing the intervention, and the broader perspectives of people providing supported for their loved ones undergoing an intervention |
|  | Should I use either descriptive or semi-structured questions? | Semi-structured interviews. Development of the interview questions required the creation of operational definitions, supported by my specialist clinical knowledge of this patient cohort and knowledge gained through my literature review. Also, the expert knowledge of author (JG) who specialises in qualitative research design, conduct and analysis. |
|  | Self-awareness: what are my skills as a researcher? | Novice qualitative researcher – limited interview skills at the start of the study, but with training developed qualitative research experience and skills over time with support and guidance from senior qualitative researchers. |
|  | How do I pre-test my data collection method? | Completed several practice interviews with healthcare professionals who have experience providing care for AS patients and their carers. Reviewed first few interviews with the research team to evaluate interview style and questions, refined questions – added hint questions to be used as needed in future interviews. |
| Sampling strategy | What is the best sampling method for my study? | Purposive sampling guided recruitment of participants (patients and carers) in the qualitative study to ensure maximum variation of participants e.g., age, gender, carer relationship. |
|  | Who are the best informants for my study? | People living with AS, and their carers.  Healthcare professionals who provide care and support for people with AS.  Qualitative research experts. |
|  | What criteria should be used to select the participants? | Condition  Criteria relevant to the questions under investigation  Inclusive – maximum variation  Diversity and representativeness of the sample |
|  | Is my sample appropriate? | The total sample size was sufficient and appropriate, more carer participants would have enabled better exploration of some minor themes  Broad diversity and inclusion approaches to study design and eligibility criteria would have resulted in a study population more representative of the Australian population. |
|  | Is my data well saturated? | Yes |
| Selecting the unit of analysis | What is the unit of analysis? | Sentence portion of paragraph analysed against operational definitions of the framework. |
|  | Is the unit of analysis too narrow or too broad? | No, it enabled effective analysis using operational definitions of the framework. |
| **Organization phase** | | |
| Categorization and abstraction | How should the concepts or categories be created? | The Theoretical Framework of Acceptability was used as a model.  To enhance the reliability of findings, data analysis included two members of the research team (NS, JG). A deductive data analysis approach was applied first, sorting findings into the core concepts of the theoretical model, cross-checking quotes against the operational definitions of the model. Data which did not completely align with the TFA’s pre-defined categories was separated and reviewed separately for inductive identification of additional themes and relevance to the research questions. Representativeness was assessed by identifying and grouping common inductive subtheme agreements (e.g., getting back to normal), with researchers refining the emerging findings by consensus. Conversely for minor themes, researchers reviewed and analysed the findings to decide if the data had sufficient weight to include. |
|  | Is there still too many concepts? | No. |
|  | Is there any overlap between categories? | In some cases, resolved as above. |
| Interpretation | What is the degree of interpretation in the analysis? | Conformability was supported by both researchers (NS, JG) independently reviewing the transcripts and then collaborating on the analysis. An additional researcher (RG) later rechecked the analysis process and findings, using the raw transcript data as a reference point to confirm accuracy. |
|  | How do I ensure that the data accurately represent the information that the participants provided? |  |
| Representativeness | How to I check the trustworthiness of the analysis process? |  |
|  | How do I check the representativeness of the data as a whole? | Cross-reference with comparable research/evidence in this field. |
| **Reporting phase** | | |
| Reporting results | Are the results reported systematically and logically?  How are connections between the data and results reported? | Reporting of the qualitative process and outcomes was closely monitored to ensure the results were described systematically and logically. The Theoretical Framework of Acceptability was used as an analytic lens to analyse the data obtained in each domain both as individual and collective interconnected components. The transferability of the results was supported by the pragmatic design of the study (e.g., aligned with routine care pathway) and the transferability of the findings to similar with similar characteristics undergoing TAVI across Australia was supported by a clear explanation of the sample and setting. |
|  | Is the content and structure of concepts presented in a clear and understandable way? | Yes, validated when preliminary results shared with broader scientific audiences and consumers |
|  | Can the reader evaluate the transferability of the results (are the data, sampling method, and participants described in a detailed manner)? | Yes. |
|  | Are quotations used systematically? | Yes. |
|  | How well do the categories cover the data? | The data fits the TFA much more accurately that the original framework. |
|  | Are there similarities within and differences between categories? | Yes, but operational definitions of the framework domains helped guide accuracy to manage similarities and differences. |
| Reporting analysis process | Is there a full description of the analysis process? | Yes. |
|  | Is the trustworthiness of the content analysis discussed based on some criteria? | Yes. |
