# Supplementary material for: A Descriptive Qualitative Study of Patient and Carer Perspectives on the Acceptability of Transcatheter Aortic Valve Implantation
Source: J Adv Nurs. 2025 Sep 3;82(5):5305–18. doi: 10.1111/jan.70180 (PMC13069208; doi:10.1111/jan.70180)
Supplement: Supplementary file 2 — Table S2: jan70180‐sup‐0002‐TableS2.docx. [file JAN-82-5305-s002.docx]

# Supplementary File 2 COREQ Checklist

Table : Consolidated criteria for reporting qualitative studies (COREQ): 32-item checklist

| **Item** | **Guide questions/Description** | **Location in Manuscript** |
| --- | --- | --- |
| **Domain 1: Research team and reflexivity** | | |
| **Personal characteristics** | | |
| 1. Interviewer/facilitator | Which author/s conducted the interview or focus group? | NS |
| 1. Credentials | What were the researcher’s credentials? E.g. PhD, MD | Title page |
| 1. Occupation | What was their occupation at the time of the study? | Title page |
| 1. Gender | Was the researcher male or female? | Female |
| 1. Experience and training | What experience or training did the researcher have? | Supplementary File 1 |
| **Relationship with participants** | | |
| 1. Relationship established | Was a relationship established prior to study commencement? | p.6 |
| 1. Participant knowledge of the interviewer | What did the participants know about the researcher? e.g. personal goals, reasons for doing the  research | Described in participant information sheet. |
| 1. Interviewer characteristics | What characteristics were reported about the interviewer/facilitator? e.g. Bias, assumptions,  reasons and interests in the research topic | Not described, implied.  Supplementary File 1 |
| **Domain 2: Study design** | | |
| **Theoretical framework** | | |
| 1. Methodological orientation and Theory | What methodological orientation was stated to underpin the study? e.g. grounded theory,  discourse analysis, ethnography, phenomenology, content analysis | p.5  deductive content analysis |
| **Participant selection** | | |
| 1. Sampling | How were participants selected? e.g. purposive, convenience, consecutive, snowball | p.6 |
| 1. Method of approach | How were participants approached? e.g. face-to-face, telephone, mail, email | p.6 |
| 1. Sample size | How many participants were in the study? | P9, Table 1. |
| 1. Non-participation | How many people refused to participate or dropped out? Reasons? | Not documented |
| **Setting** | | |
| 1. Setting of data collection | Where was the data collected? e.g. home, clinic, workplace | p.6 |
| 1. Presence of non-participants | Was anyone else present besides the participants and researchers? | No |
| 1. Description of sample | What are the important characteristics of the sample? e.g. demographic data, date | Table 1, p11. |
| **Data collection** | | |
| 1. Interview guide | Were questions, prompts, guides provided by the authors? Was it pilot tested? | Table 1, p.10 |
| 1. Repeat interviews | Were repeat interviews carried out? If yes, how many? | Not applicable |
| 1. Audio/visual recording | Did the research use audio or visual recording to collect the data? | p.6 |
| 1. Field notes | Were field notes made during and/or after the interview or focus group? | No |
| 1. Duration | What was the duration of the interviews or focus group? | 30-45mins |
| 1. Data saturation | Was data saturation discussed? | Theme coding created 100% saturation. |
| 1. Transcripts returned | Were transcripts returned to participants for comment and/or correction? | No |
| **Domain 3: Analysis and findings** | | |
| **Data analysis** | | |
| 1. Number of data coders | How many data coders coded the data? | NS, JG |
| 1. Description of the coding tree | Did authors provide a description of the coding tree? | Figure 1,2. |
| 1. Derivation of themes | Were themes identified in advance or derived from the data? | No, derived. |
| 1. Software | What software, if applicable, was used to manage the data? | p.8 |
| 1. Participant checking | Did participants provide feedback on the findings? | No. |
| **Reporting** | | |
| 1. Quotations presented | Were participant quotations presented to illustrate the themes / findings? Was each  quotation identified? e.g. participant number | Yes |
| 1. Data and findings consistent | Was there consistency between the data presented and the findings? | Yes |
| 1. Clarity of major themes | Were major themes clearly presented in the findings? | Figure 2 |
| 1. Clarity of minor themes | Is there a description of diverse cases or discussion of minor themes? | Diverse perspectives were embedded within quotes, pgs. 9-21 |
